# RETRACTION: Isolation of Antidiabetic Principle from Fruit Rinds of *Punica granatum*

**DOI:** 10.1155/ecam/9838031

**Published:** 2026-03-12

**Authors:** Evidence-Based Complementary and Alternative Medicine

RETRACTION: V. Jain, G. L. Viswanatha, D. Manohar, and H. N. Shivaprasad, “Isolation of Antidiabetic Principle from Fruit Rinds of *Punica granatum*,” *Evidence-Based Complementary and Alternative Medicine* 2012, no. 1 (2012): 147202, https://doi.org/10.1155/2012/147202.

The above article, published online on 31 July 2012 in Wiley Online Library (https://www.wileyonlinelibrary.com), has been retracted by John Wiley & Sons Ltd. The retraction has been agreed due to figure concerns initially identified on PubPeer [1]. Specifically, in Figure 5, images C and E appear to have been duplicated, resized, and rotated, despite the different experimental conditions indicated. The results and conclusions are therefore considered unreliable.

The authors did not respond when notified of the retraction.
